# Idarubicin-bromelain combination sensitizes cancer cells to conventional chemotherapy

**DOI:** 10.22038/ijbms.2019.37884.9003

**Published:** 2019-10

**Authors:** Abdullah Taşkın, Mehmet Tarakçıoğlu, Hasan Ulusal, Mustafa Örkmez, Seyithan Taysı

**Affiliations:** 1 Nutrition and Dietetics Department, Faculty of Health Science, Harran University, Şanlıurfa, Turkey; 2 Department of Biochemistry, Medical Faculty, Gaziantep University, Gaziantep, Turkey

**Keywords:** Apotosis, Bromelain, Cell survival, DNA damage, Drug interactions, Idarubicin

## Abstract

**Objective(s):**

The primary cytotoxic effects of anticancer drugs like idarubicin, a chemotherapeutic agent, are not limited to neoplastic cells; they also produce similar effects in normal cells. In this study, we hypothesized that the combination of idarubicin-bromelain could make cancer cells more susceptible to cytotoxicity and genotoxicity.

**Materials and Methods:**

To test our hypothesis, the optimal concentrations of idarubicin and bromelain were combined and incubated in the HL-60 cancer cell line and normal human mononuclear leukocytes (PBMC) for 24, 48, and 72 hr. Cytotoxicity and genotoxicity were evaluated by measurement of ATP cell viability test, DNA damage, Caspase-3, Acridine orange/ethidium bromide (AO/EB), and DAPI fluorescent dyes in both cell types.

**Results:**

The combination of idarubicin-bromelain significantly reduced cell proliferation in the more potent HL-60 compared to PBMC in all incubation times (*P<*0.05). DNA damage and Caspase-3 levels (except for 24 hr) were also higher in the HL-60 cell line in comparison with PBMC and were statistically significant (*P<*0.05). The percentages of apoptotic images obtained by DAPI and AO / EB morphological examination were increased in both cells, depending on the combination dose.

**Conclusion:**

Based on these results, it can be concluded that idarubicin combined with bromelain produces more cytotoxic effects in low concentrations in comparison with when it was used per se in the HL-60 cells. Conversely, it was found that this combination in PBMC caused less cytotoxicity and less genotoxicity. Taken together, it can be said that this new combination makes cancer cells more sensitive to conventional therapy.

## Introduction

**Figure 1:**
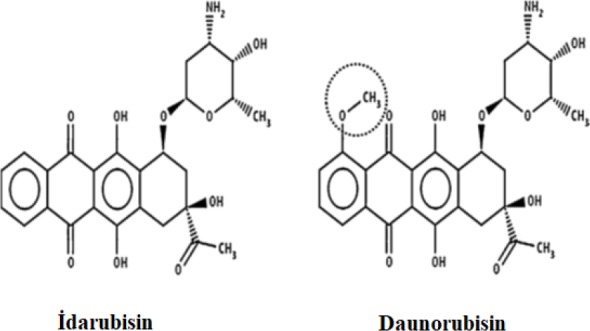
The structure of IDA and daunorubicin (7)

The formation of leukemia is a complicated process that is defined by the separation of differentiation anomalies and proliferation, resulting in leukemic clonal expansion and a block in maturation (1). IDA, which was developed against acute leukemias and certain other hematological malignancies, is a second-generation anthracycline analog that possesses less cardiotoxic effects and more anti-tumor activity in comparison with other first-generation anthracyclines (doxorubicin and daunorubicin) (Figure 1) (2). Various mechanisms regarding IDA’s primary cytotoxic effect are postulated. These theories include DNA intercalation, free radical formation, lipid peroxidation, inhibition of macromolecular biosynthesis or induction of DNA damage, DNA crosslinks and alkylation, the separation, packing, and interference in the helicase activity in the DNA helix structure, topoisomerase II inhibition and as a result, induction of apoptosis (3-5). IDA can also produce reactive oxygen species in normal cells that cause oxidative stress, especially in tissues with few free radical scavengers (6). The primary target of many anti-cancer drugs, like IDA, is nuclear DNA and to direct the cell to death processes by inflicting effective damage. The effects of IDA are not limited to neoplastic cells. It produces similar effects in normal cells, causing clinical complications in the advanced stages of chemotherapy, and so, it is used in clinical oncology according to benefit-risk analysis.

Phytochemicals have been receiving increasing attention, especially in medical science, because of their potential to protect from life-threatening diseases (8, 9). BRO, which is an extract of pineapple, (*Ananas comosus *L*., *family* Bromeliaceae*), is a phytochemical-including mixture of proteolytic enzymes and non-protease constituents. BRO is defined as a medical component because of its anti-carcinogenic effects, inhibition of platelet aggregation, fibrinolytic activity, anti-inflammatory effects, cytokine and immune system regulatory effect, drug absorption enhancing effect, mucolytic effect, digestive effect, wound healing effect, and regulatory effects in the hemostatic system (10-12). The above-mentioned effects of BRO are attributed to the presence of intrinsic protease components. The notion that BRO shows anti-cancer activity comes from traditional observations made in Southeast Asia and pre-clinical and clinical studies (10, 13).

In the present study, we investigated the effects of bromelain on idarubicin cytotoxicity in an HL-60 acute promyelocytic leukemia cell line and a culture of normal human PBMC.

## Materials and Methods

This study was conducted in the Department of Biochemistry, Medical Faculty, University of Gaziantep, Turkey, between October 2016 and August 2018. Manuscript drafting was performed in the Health Science Faculty, University of Harran, Turkey.

***Chemicals and reagents***

All chemical used for these experiments were of analytical grade. Idarubicin HCI (4-demethoxydaunorubicin) was purchased from Selleckchem (Houston, TX, USA). Bromelain, DMSO, and, unless mentioned otherwise, other chemicals were purchased from Sigma-Aldrich (St. Louis, MO, USA). RPMI 1640 was purchased from Gibco (Loughborough, Leicestershire, UK). The Cell Titer-Glo™ Luminescent cell viability assay kit was purchased from Promega (Madison, WI, USA). Caspase-3 Human Instant ELISA™ Kit was purchased from Thermo Fisher Scientific (Vienna, Austria).

***Drug preparation***

BRO solutions of different concentrations were prepared according to literature (14, 15). Final concentrations of BRO (dissolved in PBS) were 20, 10, 5, 2.5, 1, 0.5, 0.1, and 0.01 mg/ml. IDA test solutions of different concentrations were prepared as described previously (16-19). Final concentrations of IDA solubilized in DMSO were 20, 10, 5, 2.5, 1, 0.5, 0.25, 0.1, 0.05, and 0.01 μM. The final concentration of DMSO in the diluted IDA solutions was kept <1%. 

***Isolation of human PBMC and leukemia cells***

The human peripheral blood sample was obtained from a young (28 years old), healthy, non-smoking volunteer. Healthy volunteers provided informed, written consent before participating. The study was approved by the Ethical Committee of Clinical Research of the University of Gaziantep, and it was conducted in accordance with the Declaration of Helsinki. (Ethics Committee Decision Number: 2017/13, 25.01.2017). PBMC separation processes and live cell count experiments were carried out as described previously (20). The HL-60 leukemia cancer cell line (ATCC® CCL-240™) was provided by ATCC (Manassas, VA, USA). Cultures with cell viability greater than 90–95% were included in the study.

***Cell viability assay and composition of IDA-BRO combination***

The CellTiter-Glo Luminescent Cell Viability Assay was utilized to determine cell viability in HL-60 and PBMC cells. Cells were treated with or without different concentrations of BRO, IDA, and their combinations (Table 1). After 24 hr, 48 hr, and 72 hr of incubation at 37 ^°^C, assay reagent was added into each well. The luminescent signals were determined by a luminometer (Synergy H1 Multi-Mode Reader, BioTek Instruments; Winooski, VT, USA). The IC_50_ values of incubation times (24, 48, and 72 hr) were calculated separately. After carrying out the cell viability assay, three different doses of BRO and three different doses of IDA were chosen to include doses above and below the IC_50_ values determined for the PBMC and HL-60 cell lines and the combinations were created. All experiments were replicated three times.

***DNA damage determination by alkaline comet assay***

DNA damage detection in the HL-60 cell line and PBMC was performed by using the alkaline single-cell gel electrophoresis assay method (comet assay) developed by Singh *et al*. with minor modifications (21, 22). The HL-60 cell line and PBMC were placed into 6-well cell culture plates containing RPMI 1640 medium and incubated for 24 hr at 37 ^°^C in 5% CO_2_. IDA-BRO combinations and specific IDA concentrations for each incubation period were treated with both HL-60 and PBMC cells and incubated for 24, 48, and 72 hr. Following each incubation period, DNA damage experiments and assessments were carried out as described previously (23). The experiments were repeated three times.

***Determination of caspase-3 assay***

A Caspase-3 assay was performed to investigate the apoptotic activity of IDA and IDA-BRO combinations in the HL-60 cell line and PBMC. Caspase-3 levels were measured using a commercial ThermoFisher Scientific Caspase-3 Human Instant ELISA Kit. The test samples were run in duplicate as three replicates. The results were expressed in ng/ml.

***AO/EB fluorescent staining***

AO/EB fluorescent staining to assess morphological differences in the HL-60 cell line and PBMC was performed as described in literature (24). After the incubation periods, nuclear morphology was assessed by fluorescence microscopy (Leica, DM IL LED, Wetzlar, Germany). As described elsewhere, 100 images randomly selected from each sample were visually analyzed (25). All experiments were performed three times.

***DAPI fluorescence staining***

The fluorescence intensity, as a result of nuclear fragmentation and chromatin condensation in the detection of apoptotic cells by DAPI staining, is stronger than that of normal cells. After the incubation periods, the DAPI fluorescence staining method was carried out as described previously (26). The morphologies of stained samples were examined, and 100 cells were counted by a fluorescence microscope (Leica, DM IL LED, Wetzlar, Germany). Experiments were repeated three times. 

***Statistical analysis***

Statistical analyses were performed using SPSS for Windows, Version 20.0 software (IBM SPSS Inc, Chicago, IL, USA). The results are represented as a mean±standard deviation. Data in all experiments were analyzed for statistical significance using analyses of variance (Kruskal Wallis-H). A Mann-Whitney U test was used to compare the two groups. The relationship between the parameters was investigated by a Spearman correlation analysis. A *P-value<0.05* was considered statistically significant.

## Results

***Cell viability assay***

**Figure 2:**
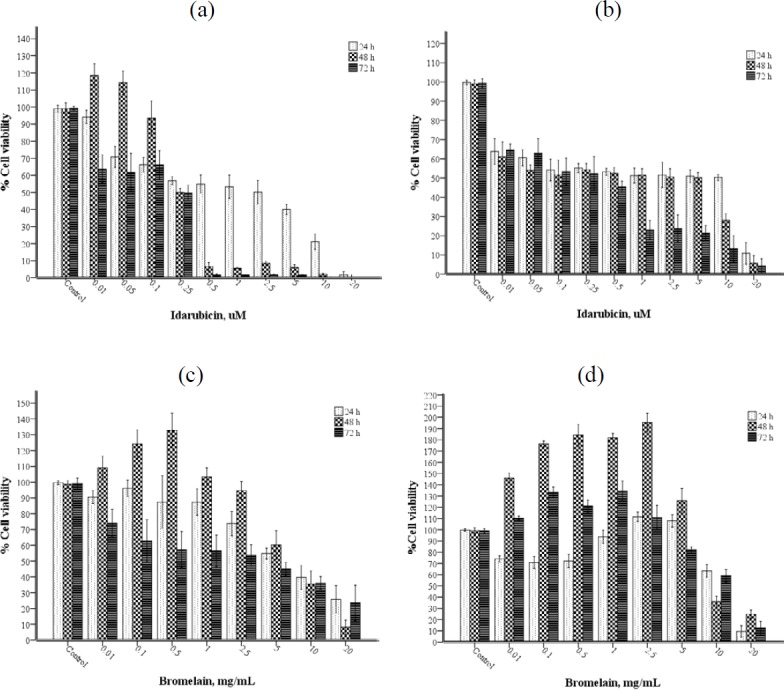
(a-d) Effect of IDA and BRO on HL-60 (a, c) and PBMC (b, d) after 24, 48, and 72 hr incubation. Data are representative of three independent experiments

The IC_50_ values of IDA (0.01-20 μM) and BRO (0.01-20 mg/ml) were determined separately for each cell to generate the combination (Figure 2). The IC_50_ values of IDA after 24, 48, and 72-hr incubation periods in HL-60 and PBMC were calculated as 2.5, 0.25, 0.25; and 10, 7, and 0.5 μM, respectively. The IC_50_ values of BRO after incubation periods in HL-60 and PBMC were calculated as 7.5, 7.5, and 2.5; and 12, 9, and 11 μM, respectively.

**Figure 3:**
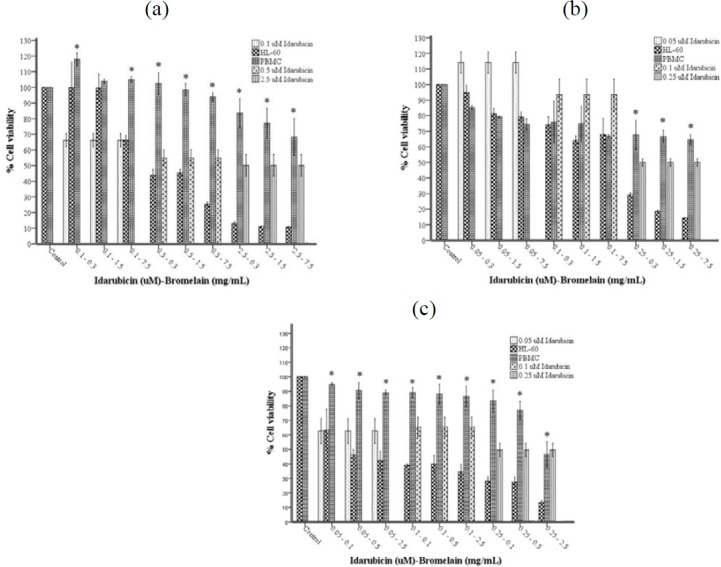
(a-c). Growth inhibition of PBMC and HL-60 cell lines treated with IDA-BRO combinations by The CellTiter-Glo Luminescent Cell Viability Assay after 24 (a), 48 (b), and 72 (c) hr incubation. Each point represents a mean±SD of three experiments with three replicates per combination. Values marked with * indicate significant differences between HL-60 and PBMC for the same combination

IDA-BRO combinations were made according to IC_50_ values calculated separately for HL-60 and PBMC (Table 1). At the end of almost all incubation times, the cytotoxic effect of all combinations on the HL-60 cell line was found to be higher than PBMC (Figure 3). The IDA-BRO combination made HL-60 cells more susceptible to cytotoxicity according to the single concentration of IDA.

***Genotoxic assay***

**Figure 4:**
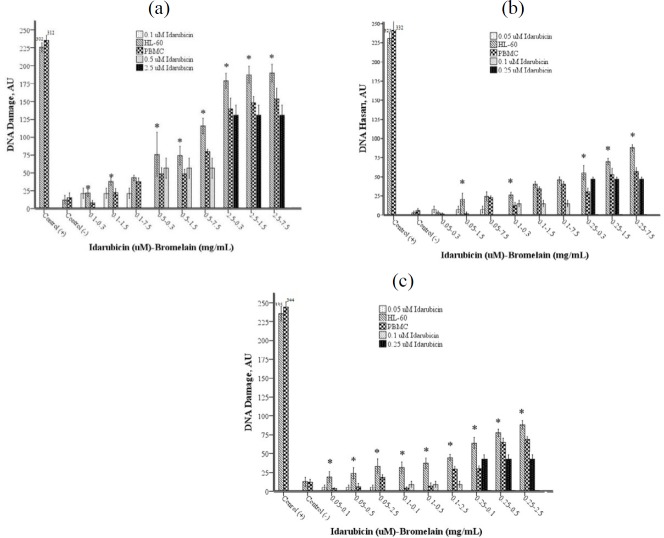
(a-c). DNA damage levels in the HL-60 cell line and PBMC after 24 (a), 48 (b), and 72 (c) hr incubation. Each point represents a mean±SD of three experiments with two replicates per combination. The levels of DNA damage of HL-60 are significantly (*) different (*P<*0.05) from PBMC at the same combination

The genotoxic effects of the combinations were examined with the comet assay method. DNA damage increased concentration-dependently in both cell groups at the end of all three incubation periods. The DNA damage in the HL-60 cell line was found to be higher compared to PBMC, with a few exceptions (Figure 4).

**Figure 5:**
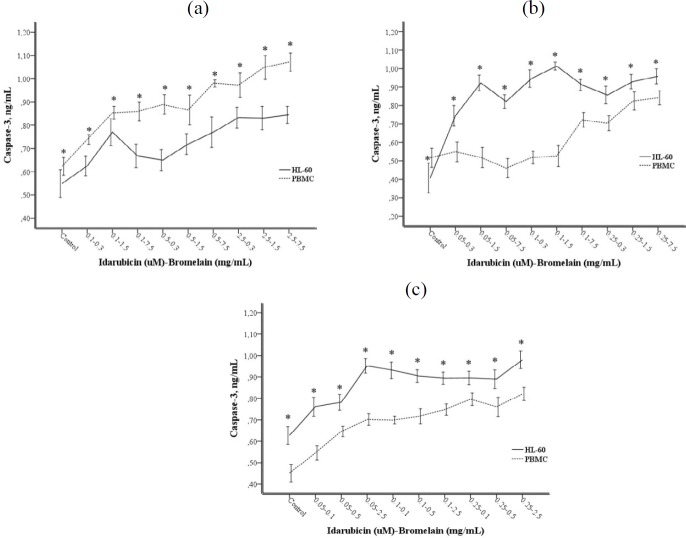
(a-c). Caspase-3 levels after 24 (a), 48 (b), and 72 (c) hr incubation. Each point represents a mean±SD of three experiments with three replicates per combination. Values marked with * indicate significant differences between HL-60 and PBMC at the same combination (*P<*0.05)

**Figure 6:**
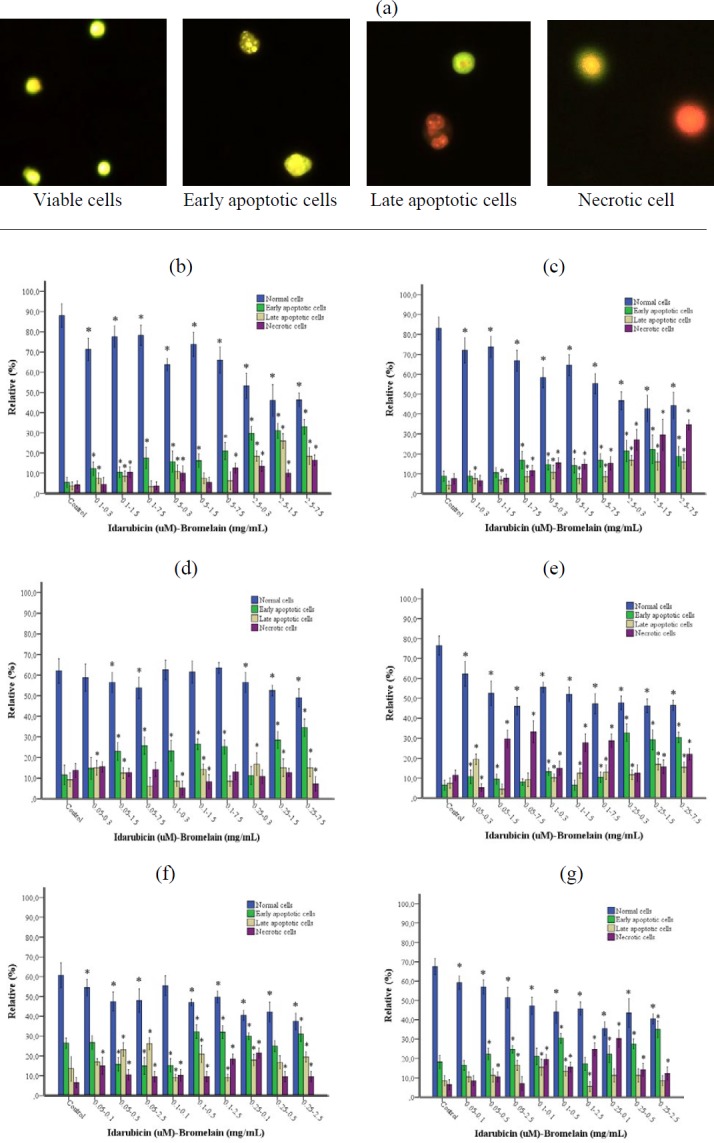
(a-g). Detection of apoptosis by AO/EB dual staining. (a) Viable cells, early apoptotic cells, late apoptotic cells, and necrotic cells were detected using fluorescence microscope observation following AO/EB staining. Magnification was 40X. Significant changes were observed in HL-60 and PBMC exposed to IDA-BRO combinations at 24 (b and c), 48 (d and e), and 72 (f and g) hr incubation, respectively. Each column represents a mean±SD of three experiments with three replicates per combination. *P<0.05 vs Control group (HL-60 or PBMC)

**Figure 7:**
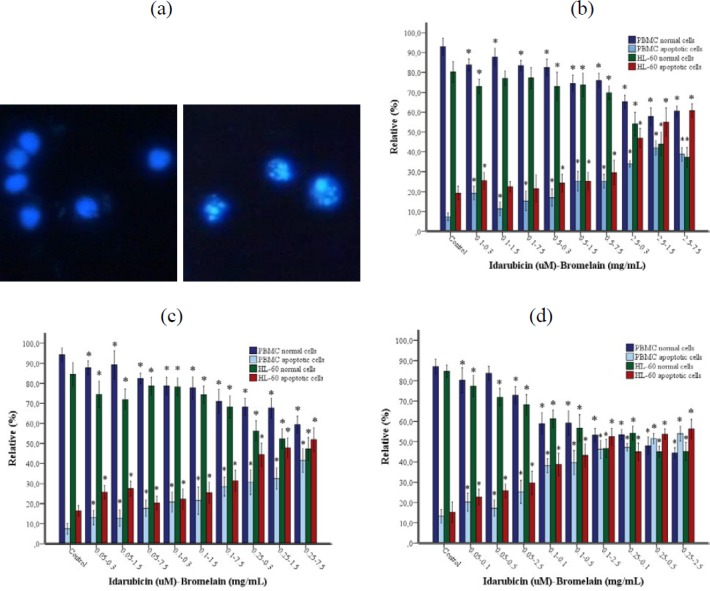
(a-d). Apoptotic and normal cell percentages determined by DAPI fluorescence staining method. (a) Normal cells and apoptotic cells. Magnification was 40X. (b) 24, (c) 48, (d) 72 hr incubation. Each column represents a mean±SD of three experiments with three replicates per combination. *P<0.05 vs Control group (HL-60 or PBMC)

**Table 1:** IDA and BRO concentrations used in single agent and combination treatment of PBMC and HL-60 cell lines

**Time**	**Single agent IDA (μM) / ** **BRO (mg/mL)**	**Combinations [IDA(μM) / BRO (mg/mL)]**
**24 hr** **48 hr** **72 hr**	20 10 5 2.5 1 0.5 0.25 0.1 0.05 0.01 20 10 5 2.5 1 0.5 0.1 0.01 20 10 5 2.5 1 0.5 0.25 0.1 0.05 0.01 20 10 5 2.5 1 0.5 0.1 0.01 20 10 5 2.5 1 0.5 0.25 0.1 0.05 0.01 20 10 5 2.5 1 0.5 0.1 0.01	0.1/0.3 0.1/1.5 0.1/7.5 0.5/0.3 0.5/1.5 0.5/7.5 2.5/0.3 2.5/1.5 2.5/7.5 0.05/0.3 0.05/1.5 0.05/7.5 0.1/0.3 0.1/1.5 0.1/7.5 0.25/0.3 0.25/1.5 0.25/7.5 0.05/0.1 0.05/0.5 0.05/2.5 0.1/0.1 0.1/0.5 0.1/2.5 0.25/0.1 0.25/0.5 0.25/2.5

***Caspase-3 assay***

The caspase-3 levels were found to be as high as DNA damage and cytotoxicity at 48 and 72 hr incubation times in the HL-60 cell line as compared to PBMC (Figure 5). The high caspase-3 levels, which correlate to an increase in the concentration of IDA-BRO, are indicative of cell death by apoptosis.

***AO/EB fluorescent staining***

It was found that while the early apoptotic, late apoptotic, and necrotic cell rates in the IDA-BRO combinations during three incubation periods in both cell lines increased in a concentration-dependent fashion, the normal cell rate decreased and was statistically significant (*P<0.05*). These results revealed morphological changes and shrinkage of cells leading to cell apoptosis included by the IDA-BRO combination in human PBMC and HL-60 cell lines.

***DAPI fluorescence staining***

The fluorescence intensity, as a result of nuclear fragmentation and chromatin condensation in the detection of apoptotic cells by DAPI staining, is stronger than that of normal cells (Figure 7). It was found that during all three incubation periods, the apoptotic cell rate increased with IDA/BRO concentration, while the normal cell rate decreased and was statistically significant (*P<0.05*).

## Discussion

The balance between the therapeutic and toxicologic effects of a chemical compound becomes an important parameter in the pharmacological applicability of that component (27). Many cytotoxic drugs activate apoptosis or other cell death mechanisms by inducing or inhibiting critical intracellular signaling pathways by means of killing malignant cells (28). In this study, we investigated the cytotoxic and genotoxic effects of idarubicin and bromelain on a human acute promyelocytic leukemia cell line (HL-60) and normal human mononuclear lymphocyte cells by creating different combinations of the two agents.

Many studies of cancer cells show that the combined use of anti-carcinogenic materials and chemotherapy agents that are already used generally generates additive and synergistic effects (29, 30). In cancer treatment, the toxicity of cytotoxic and cytostatic drugs to normal hematopoietic bone marrow cells is generally a dose-limiting factor. This toxicity can cause death as a result of infection or bleeding. In addition, it is theorized that too much bone marrow toxicity can cause non-optimal treatment of the tumor. Furthermore, the genotoxic effect of anticancer drugs on normal cells can cause the induction of secondary malignancies or further worsen the disease. For this reason, the inhibition of such a mechanism could significantly increase survival rates.

In our study, we found that an IDA-BRO combination concentration-dependently generated more cytotoxic effects on the HL-60 cell line compared to PBMC after 24, 48, and 72-hr incubation periods. The cytotoxic effect of IDA/BRO combinations in the HL-60 cell line in all of the incubation periods was higher than the cytotoxic effect created by a single concentration of IDA. The fact that the cytotoxic concentrations formed in the HL-60 cell line by idarubicin, which executes its cytotoxic effect by inducing cell death processes through nuclear DNA or by causing intracellular free radical formation, were not seen in PBMC suggests that this is caused by antioxidant defense systems and DNA repair mechanisms in the lymphocyte cell culture. These results are important findings in terms of showing the necessity for higher idarubicin concentrations in order to create cytotoxic effects in lymphocyte cells as compared with HL-60 cells.

There is a linear correlation between DNA damage and cell death in high concentrations of idarubicin (16, 17). Some studies show a positive correlation between the amount of DNA damage and cell death, and it was stated that DNA damage is decisive for cytotoxicity (31, 32). In this study, it was found that the level of DNA damage increased with IDA/BRO concentrations in both cell lines at the end of three incubation periods. At the end of all three incubation periods, the level of DNA damage created in the HL-60 cell line by all combinations was found to be higher as compared to PBMC. We believe that the cytotoxic effects created by idarubicin can be compensated with the fact that the regulatory effects and the DNA damage levels of bromelain are on a tolerable level in PBMC and accordingly, with the activation of DNA repair mechanisms. However, it is suggested that in the HL-60 cell line, the failure of repair mechanisms to be activated as a result of the genomic instability occurred due to high levels of DNA damage or defects in the genes that encode DNA repair enzymes reduce cell viability by inducing cell death processes.

Studies report that cytotoxic agents causing DNA damage also activated apoptosis (33, 34). Genetic and biochemical data show that caspases play an important role in the conduction phase of apoptosis (35). In a study conducted by Qi *et al*., it was reported that idarubicin induced the apoptotic pathway through caspase-3/7 in Jurkat leukemia cells and HL-60 (36). In this study, the caspase-3 level in the HL-60 cell line was higher compared to PBMC at 48 and 72 hr incubations, but not the 24 hr incubation period. As the IDA-BRO concentration increased, the caspase-3 level also increased proportionally. 

Apoptosis can be measured with different methods based on morphological, biochemical, and molecular changes that occur in a cell (37). In this study, we evaluated apoptosis in three different ways: Caspase-3 and morphological examination via the fluorescent microscope using AO/B and DAPI fluorescent staining methods. The results of the existing study showed that the cancer cells treated/cured with the IDA-BRO combination showed a significant dose-based reduction in cell viability and more apoptotic views (chromatin condensation and nuclear fragmentation) when compared with normal cells. Interestingly, it was found with AO/EB morphological examination that the necrotic cell rate was higher in PBMC as compared to the HL-60 cell line. When the HL-60 cell line and PBMC were evaluated in each incubation period, it was seen that there was a positive correlation between DNA damage and Caspase-3 level and fluorescent staining methods, while there was a negative relationship to cell viability.

## Conclusion

At the conclusion of this study, it was observed that the IDA-BRO combination had more cytotoxic and genotoxic effects on the HL-60 cell line as compared to PBMC. Of course, this does not tell us that the IDA-BRO combination definitely selectively targets leukemia cells; however, it furthers the view that idarubicin combined with bromelain creates more cytotoxic effects in lower concentrations compared to when it is used by itself and that the same combination can create less cytotoxic effects in normal cells.

The findings of our research may be a guide for the development of new approaches, identification of new molecules and *in vivo* and clinical studies of the use of bromelain preparations for the treatment of leukemia. Also, our proof-of-concept studies could potentially be extended to other cell cultures, and these mechanism-based, cytotoxicity-decreased strategies (for normal cells) could help optimize conventional chemotherapy further.
